# Meta‐Transcriptomes From Microcosms From a Cr Impacted Soil Provides Insights Into the Metabolic Response of the Microbial Populations to Acetate Stimulation

**DOI:** 10.1111/1758-2229.70148

**Published:** 2025-07-07

**Authors:** Douglas I. Stewart, Elton J. R. Vasconcelos, Ian T. Burke, Alison Baker

**Affiliations:** ^1^ School of Civil Engineering University of Leeds Leeds UK; ^2^ Leeds Omics University of Leeds Leeds UK; ^3^ School of Earth and Environment University of Leeds Leeds UK; ^4^ School of Molecular and Cellular Biology University of Leeds Leeds UK

**Keywords:** acetate, alkaline environment, bioremediation, chromium, COPR, meta‐transcriptome

## Abstract

Environmental contamination by Cr(VI) leaching from chromite ore processing residue (COPR) legacy disposal sites can pose a threat to human health. Under iron‐reducing conditions, microbial activity can convert mobile and toxic Cr(VI) to less mobile and less toxic Cr(III); however, COPR waste is a very hostile environment for microbial life. Microcosms using soil from beneath a COPR disposal site were challenged with Cr(VI) with and without acetate to stimulate microbial metabolism. Geochemistry showed that when the microbial populations were reducing iron, Cr(VI) was also reduced, and 16S rRNA gene sequencing showed that the community composition evolved over the course of the experiment. Meta‐transcriptome data revealed ~3% of transcripts were differentially regulated (*p* = 0.01) between the acetate amended and unamended systems, with twice as many transcripts downregulated by acetate. Gene ontology (GO) terms for processes involving the cell wall, cell periphery, plasma membrane and encapsulating structures as well as catabolic processes, especially carbohydrate metabolism, were significantly enriched in the unamended microcosm meta‐transcriptome. Transcripts for alternative sigma (σ) factors and anti‐σ factors were prominent among the differentially regulated genes. The study provides insight into how the provision of acetate shapes metabolic processes and life history strategies in an alkaline Cr(VI) impacted environment.

## Graphical abstract

Soil samples from below a COPR waste tip were used to inoculate microcosms which were challenged with Cr(VI). One microcosm was also supplemented with acetate. Microbiology was sampled and bacterial gene expression analysed. Created in BioRender. Baker (2025) https://BioRender.com/j84z498.

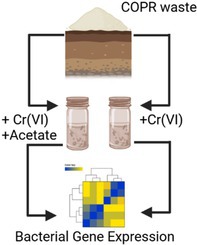

## 1 Introduction

Dumped chromite ore processing residue (COPR) at legacy waste sites poses a threat to health and the environment through the leaching of toxic Cr(VI) into groundwater. Some of the most intractable environmental problems are associated with poor disposal of COPR from the high‐lime process (Breeze 1973; Burke et al. 1991; Farmer et al. 1999). While high‐lime processing has long been superseded, its simplicity meant it continued to be widely used until the end of the 20th Century, particularly where environmental regulations were less stringent (Wang et al. 2013; Matern et al. 2016). In 2000, about a third of world production of chromate chemicals was still by the high‐lime process, producing about 600,000 t/year of contaminated waste (Darrie 2001). Rainfall ingress into this waste can produce water with a pH > 12 containing up to 80,000 μg/L Cr(VI) (Whittleston et al. 2011).

When COPR leachate escapes into the subsurface environment, reactions with soil minerals and soil organic matter can buffer the pH into the range pH 8.5–10.5 (Ding et al. 2016; Pertusatti and Prado 2007; Carroll and Walther 1990). In this pH range, soil microorganisms can reduce microbially available Fe(III) to Fe(II) (Zavarzina et al. 2006; Thorpe et al. 2012; Williamson et al. 2013; Fuller et al. 2014). Aqueous Cr(VI) will react with the microbially reduced Fe(II) and precipitate as less mobile and less toxic Cr(III) hydroxides, so iron‐reducing conditions can greatly lessen the risk of Cr contamination to the environment (Pourbaix 1966; Anderson 1997; Buerge and Hug 1999). This process has been observed at several disposal sites where COPR leachate has fortuitously seeped into organic matter‐rich iron‐reducing soils, resulting in the accumulation of chromium as the Cr(III) form associated with Fe(III) oxy‐hydroxides (Burke et al. 1991; Whittleston et al. 2011; Ding et al. 2016).

In situ bioremediation using electron donors such as ethanol or acetate has been tested extensively for uranium contaminated sites and shown to stimulate the reduction of harmful U(VI) using naturally occurring electron acceptors, such as Fe(III) (Anderson et al. 2003; Vrionis et al. 2005) with *Geobacter* spp. playing an important role (Williams et al. 2011). However, field studies are expensive to carry out, have many variables that are difficult to constrain, and face regulatory hurdles in many parts of the world. Microcosms and other culture systems derived from environmental samples, while not completely representative of the natural environment, can provide a more tractable means of exploring geochemical changes associated with microbial population evolution and metabolic processes (Stewart et al. 2024; Beller et al. 2014; Pei et al. 2020).

The aim of this study was to apply molecular tools to understand the evolution and transcriptional activity of a soil microbiome from a COPR waste site following the addition of acetate as an electron donor. This is an extreme environment for soil microorganisms (high pH and metal toxicity) that has not been well explored, and so without better understanding, it is difficult to exploit these soil microbial processes for environmental gain. Although both populations showed indicators of the general stress response, acetate partially alleviated this, presumably by alleviating carbon limitation stress. Differences in gene expression between the two systems are discussed in relation to mechanistic understanding of the effects of acetate in defined culture systems and also of models for microbial life history strategies.

## 2 Experimental Procedures

### 2.1 Site Description and Soil Sampling

Soil and water were collected from a 19th century chromium ore processing residue (COPR) tip located in a river valley in the North of England. Prior ground investigations at the tip have revealed that the COPR waste was placed directly onto the natural soil deposits at the site and covered with topsoil later. Directly beneath the tip there is a thin soil layer that is rich in soil organic matter. This has been described as a grey clay and is thought to have been the surface layer prior to COPR tipping. Alluvial soils (silt, clay and sand) underlie the grey clay. A full site description is reported by Stewart et al. (2024).

Soil samples were collected in June 2021 from a borehole that was advanced through the side‐slope on the western corner of the waste tip using a hand auger and 1 m core sampler. This borehole revealed 1.8 m of compacted topsoil, over 1.1 m of COPR waste, over grey clay soil. The soil used in this study was a sample of grey clay recovered from a depth of 2.9 m (immediately below the COPR). This soil sample was double‐bagged in sealed polythene bags and stored at 4°C in the dark in an oxygen‐free atmosphere using Anaerogen sachets. A subsample was frozen for DNA extraction and 16S rRNA gene sequencing. A water sample was taken in November 2021 from a drainage ditch at the base of the southern slope of the COPR pile and stored at 4°C in completely full sealed polythene containers.

### 2.2 Microcosm Experiments

Microcosms were made from 10 g of clay and 100 mL of ditch water in 120 mL Wheaton glass serum bottles (in November 2021). These were purged with N_2_ and sealed with butyl rubber stoppers and aluminium crimp‐seals (Merck Life Sciences, Germany). Triplicate microcosms were made for an ‘*unamended’* system (replicate numbers were prefixed with the letter A) and an *‘acetate‐amended’* system (prefixed with B). The *‘unamended’* microcosms were sealed without further addition, whereas sodium acetate to a final concentration of 20 mmol L^−1^ was added to *‘acetate‐amended’* microcosms before the bottles were sealed. Sterile controls for each system were prepared by autoclaving soil in bottles with a N_2_ purged headspace soil (121°C for 15 min) before injecting filter sterilised ditch water upon cooling. Sodium acetate was added to the acetate‐amended sterile control. All microcosms and controls were incubated anaerobically at 21°C ± 2°C in the dark.

The ditch water contained 420 mmol L^−1^ Cr(VI). The microcosms were subsequently spiked with Cr(VI) on Days 7 and 21, and on the day before sampling the microbiology. The purpose of this was to maintain the habituation of the bacterial populations to Cr. The spiking solution contained K_2_CrO_4_ to a final concentration of 500 μmol L^−1^ Cr(VI), together with NaOH to a final concentration of 7.5 μmol L^−1^ and, for the acetate‐amended microcosms, sodium acetate to a final concentration of 10 mmol L^−1^. Microcosms were periodically sub‐sampled for geochemical analysis. During sampling, the bottles were shaken, and 3 mL of soil slurry was withdrawn through the stopper using aseptic technique with sterile syringes and needles. Samples were centrifuged (5 min, 16,000 g) and the pore water and soil were analysed. The microcosm experiments were terminated, and the microbiology was sampled on either Day 43 (unamended system) or Day 49 (acetate‐amended system). The microcosms were sampled in a period when the geochemical indicators in both systems were at similar values and stable, and when the same time interval had passed after the final Cr spike was added. This produced a 6‐day difference in the overall length of incubation between the two systems but also allowed for maximising the speed of RNA recovery (as RNA degrades very rapidly in soil environments).

### 2.3 Geochemical Methods

Aqueous Cr(VI) was determined by a standardised UV–vis spectroscopy method on a Shimadzu UV‐1900 (US‐EPA 1992). Chloride, nitrite, nitrate and sulphate were determined by ion chromatography on a ThermoScientific ICS5000 with AS19 and AG19 analytical columns. As a proxy for microbial available Fe, the percentage Fe(II) in the soil was determined after extraction by 0.5 N HCl and reaction with ferrozine (Lovley and Phillips 1986). pH was determined using a Denver Instruments UB‐10 bench‐top metre and Sentek P11 pH electrode calibrated at 7 and 10, daily.

Loss on ignition (LOI) at 550°C (4 h) was determined on samples that were first oven dried at 105°C (24 h). Relative Intensity Ratio (RIR) quantitative x‐ray diffraction analysis (qXRD) was carried out on the Bruker D8 XRD using Cu Ka_1_ radiation (for details see Stewart et al. 2024). Trace elements (Fe, S and Cr) in the clay samples were determined using an Olympus Innovex X‐5000 energy dispersive X‐ray Fluorescence spectrometer (XRF).

### 2.4 Microbiological Methods

RNA was extracted from subsamples of soil (1.75 ± 0.03 g) from each microcosm immediately after termination of these experiments using a RNeasy PowerSoil Total RNA Kit (QIAGEN Ltd). The remaining soil was frozen. DNA was extracted from subsamples of the frozen soil (0.281 ± 0.040 g) using a DNeasy PowerSoil Kit (QIAGEN Ltd). DNA was quantified using a Qubit dsDNA HS Assay on a Qubit 2.0 Fluorometer (Invitrogen) and concentrations ranged from 6 to 81 ng/μL. RNA was quantified using a TapeStation (Agilent) and concentrations ranged from 13.5 to 62.7 ng/μL and RIN numbers from 7.8 to 8.9. RNA and DNA samples were sent to the Next Generation Sequencing Facility at the Leeds Institute for Biomedical and Clinical Sciences for RNA‐Seq transcriptomic analysis, whole genome sequencing, and 16S rRNA gene sequencing targeting the hyper‐variable V4 region.

### 2.5 16S rRNA Gene Microbiome Data Analysis

UPARSE pipeline (Edgar 2013) was used for the 16S rRNA sequencing analysis, setting a 97% identity threshold for clustering operational taxonomic units (OTUs). Sufficient reads were recovered from all soil samples to randomly select 14 k reads per soil sample after quality control for subsequent analysis. Taxonomic classification of OTUs was undertaken using the RDP 16S rRNA training database version 16 (Cole et al. 2014) using a confidence value of 0.7 to give a reasonable trade‐off between sensitivity and error rate in the taxonomy prediction. OTUs which were not classified as bacteria with a confidence > 0.7 (e.g., Archaea and poor reads) were not included in the diversity and statistical analyses. Hill numbers (*D*_*q*_) were used to characterise bacterial diversity in the samples (Hill 1973), where *D*_0_ is the OTU richness and *D*_1_ and *D*_2_ are measures of the number of common and dominant OTUs. Bray‐Curtis beta diversity values were used to characterise dissimilarity between the replicates.

### 2.6 Meta‐Genomics and Meta‐Transcriptomics Data Analyses

Biological triplicates from the unamended and acetate‐amended systems had both their total DNA and total RNA extracted and sequenced as described above. An average of 76.5 and 70.8 million paired‐end reads, showing high base calling quality (*Q* > 30) after trimming adapters, were obtained for both DNA‐ and RNA‐sequencing strategies. The same ad hoc MetaWRAP pipeline (Uritskiy et al. 2018) deployed previously by us (Stewart et al. 2024) was employed herein for the generation of metagenome‐assembled genomes (MAGs) from the current DNA‐seq libraries that matched the RNA‐seq samples. Whole Genome Shotgun MetaWRAP workflow consists of the following several steps: (i) quality control and trimming of reads (FastQC and TrimGalore); (ii) assembly of metagenomes (metaSpades); (iii) taxonomic classification of the assembled contigs (Kraken2); (iv) contigs' binning to generate MAGs (Concoct, MaxBin2 and metaBAT2); (v) bins' refinement and classification (checkM); (vi) functional annotation of MAGs' genes (Prokka) and KEGG Orthology assignment (prokka2kegg.py). Sequencing parameters are reported in Table S7 and the data have been deposited in ENA BioProject PRJEB51999. MAGs from unamended (A) system and acetate‐amended (B) system had their sequence (fasta) and annotation (gff) files concatenated to solely serve as a reference genome for the downstream RNA reads alignment and differential expression (DE) analyses (Table S8). RNA‐seq libraries were aligned against the MAGs with bowtie2 (Langmead and Salzberg 2012) using the parameters reported in Table S6. Samtools v1.16.1 (Li et al. 2009) was used to create sorted binary alignment map (BAM) files. These BAM files then served as input for the program featureCounts (Liao et al. 2013) in order to assign read counts to genes, using the MAGs' gff annotation files from both the unamended and acetate‐amended systems (featureCounts' parameters are reported in Table S6).

The read counts table generated by featureCounts was then used as input for DE analysis with the MTX model using the parameters reported in Table S6 (Zhang et al. 2021). An ordinary *p* value < 0.01 threshold was set on the overall MTX model DE output table for selecting differentially expressed genes (DEGs) based on the acetate‐amended system B/unamended system A contrast. The same read counts table was also submitted to a multi‐dimensional scaling (MDS) analysis using the *plotMDS* function from the EdgeR package (Robinson et al. 2009). EnhancedVolcano (Blighe et al. 2024) was employed for an overall DE visualisation through volcano plots.

Eggnog mapper v2.1.12 (Cantalapiedra et al. 2021) was run on MAG's protein‐coding genes identified by Prokka (metaWrap pipeline) to build a cluster of orthologous groups (COGs) reference database to serve as background control for downstream gene enrichment analyses. Gene ontology (GO) terms' and KEGG pathways' enrichment analyses for individual COGs lists (derived from up‐ and downregulated DEGs in the acetate‐amended system B) were performed by clusterProfiler v4.0 (Wu et al. 2021) in the R v4.3 software environment, setting the adjusted *p* value to < 0.1 through the *enricher* function. Gene expression heat maps for all DEGs, as well as for K07315‐related DEGs, were plotted through the *pheatmap* R package (Kolde 2019).

## 3 Results

The grey clay consisted predominantly of quartz with illite/smectite, some albite, and smaller amounts of calcite, mica and kaolinite (clay minerals were about 30% of the sample; Table S1). It contained 3.4 wt% Fe, 0.3 wt% S and 0.2 wt% Cr (Table S2). 44% ± 13% of acid‐extractable iron was Fe(II). LOI was 8.1 wt%, suggesting it contained about 3.0 wt% soil organic carbon, SOC (Jensen et al. 2018). The conditioned ditch water had a pH value of 9.0 and contained 420 mmol L^−1^ (22 mg/L) Cr(VI), 100 mmol L^−1^ NO_3_^−^ and 1040 mmol L^−1^ SO_4_^2−^ (Table S3).

### 3.1 Microcosm Experiments

**FIGURE 1:**
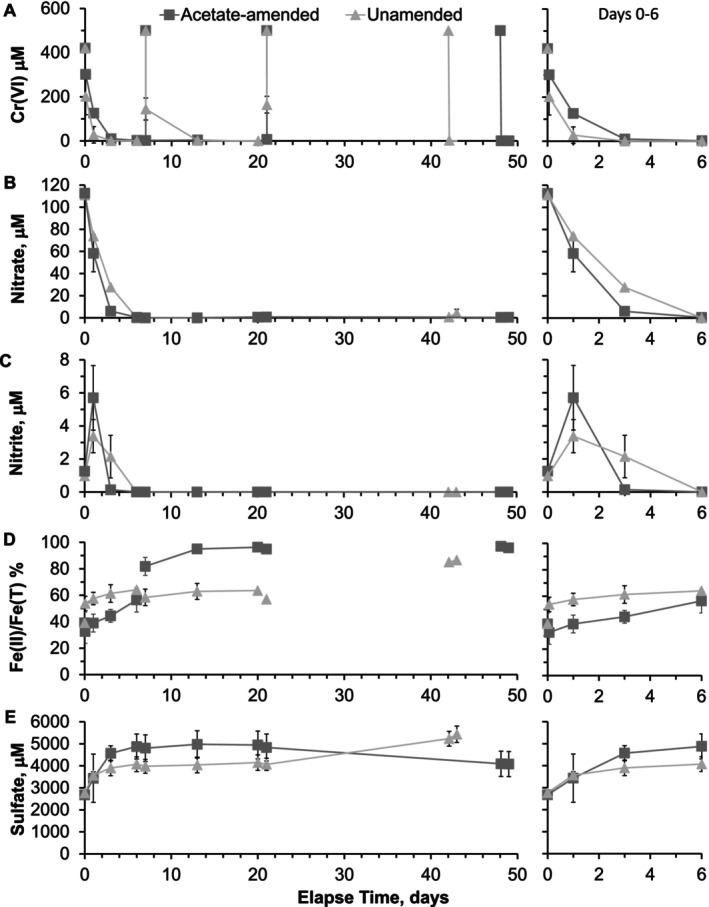
Geochemical conditions in the unamended and acetate‐amended microcosms (A) Cr(VI) concentration, (B) nitrate concentration, (C) nitrite concentration, (D) percentage of the acid extractable Fe as Fe(II), and (E) sulphate concentration. Error bars represent one standard deviation of triplicates. Panels on right‐hand side show an expanded view of Days 0–6.

The microcosms were first sampled ~90 min after establishment. At this point, the geochemistry of the two systems was very similar (Figures 1 and S1). The initial pH of both systems was 8.3 ± 0.1, but their pH varied between 8.0 and 8.6 over the course of the experiment (the pH of both systems decreased slightly with time but was restored ~pH 8.5 by the Cr(VI) spiking solution; see Figure S1A). At the first sampling point, the water contained 251 ± 76 μmol L^−1^ Cr(VI), 112 ± 3 μmol L^−1^ NO_3_^−^ and 2720 ± 80 μmol L^−1^ SO_4_^2−^. Approximately 43% ± 14% of the 0.5 N HCl extractable iron associated with the soil was Fe(II).

All the Cr(VI) introduced with the ditch water was removed from solution over about 3 days in both systems (Figure 1A). All three subsequent Cr(VI) spikes were also removed rapidly from solution in both microcosm series (Cr(VI) was removed from solution within 30 min of the second and third spike, but removal time could not be quantified for the first spike due to the sampling interval).

Nitrate was removed from both systems during the first few days, so that when the Cr(VI) was respiked on Day 7, nitrate was below the detection limit in both systems (Figure 1B). During this period, aqueous nitrite was detected in both systems, but the concentration was higher and persisted for longer in the unamended system (Figure 1C).

About half of the acid‐extractable iron was initially Fe(II) in both microcosm experiments. This proportion increased with time, to ~85% and 95% in the unamended and acetate‐amended systems at the time of sampling (Figure 1D). No systematic change in the proportion of the acid‐extractable iron that was Fe(II) was observed due to the Cr(VI) spikes.

Sulphate concentration exhibited a modestly increasing trend in both microcosm series during the first 20 days, reaching 4140 ± 350 μmol L^−1^ in the unamended microcosms and 4950 ± 630 μmol L^−1^ in the acetate‐amended microcosms (Figure 1E). Subsequently, there was a modest further increase in the sulphate concentration in the unamended microcosms to 5430 ± 370 μmol L^−1^. However, in the acetate‐amended microcosms, there was a decrease in the sulphate concentration starting after Day 20, reaching a final concentration of 4080 ± 570 μmol L^−1^ (an 18% decrease). The mean acetate concentration was 3 ± 3 μmol L^−1^ in the unamended microcosms with no systematic variation during the experiment (Figure S1B). In the acetate‐amended microcosms, the mean acetate concentration was 22.6 ± 3 mmol L^−1^ before the first Cr(VI) spike and increased to 27.4 ± 0.5, 39.4 ± 0.8 and 48.0 ± 0.1 mmol L^−1^ after the 1st, 2nd and 3rd Cr(VI) spikes.

### 3.2 16S rRNA Gene Sequence Analysis

**FIGURE 2:**
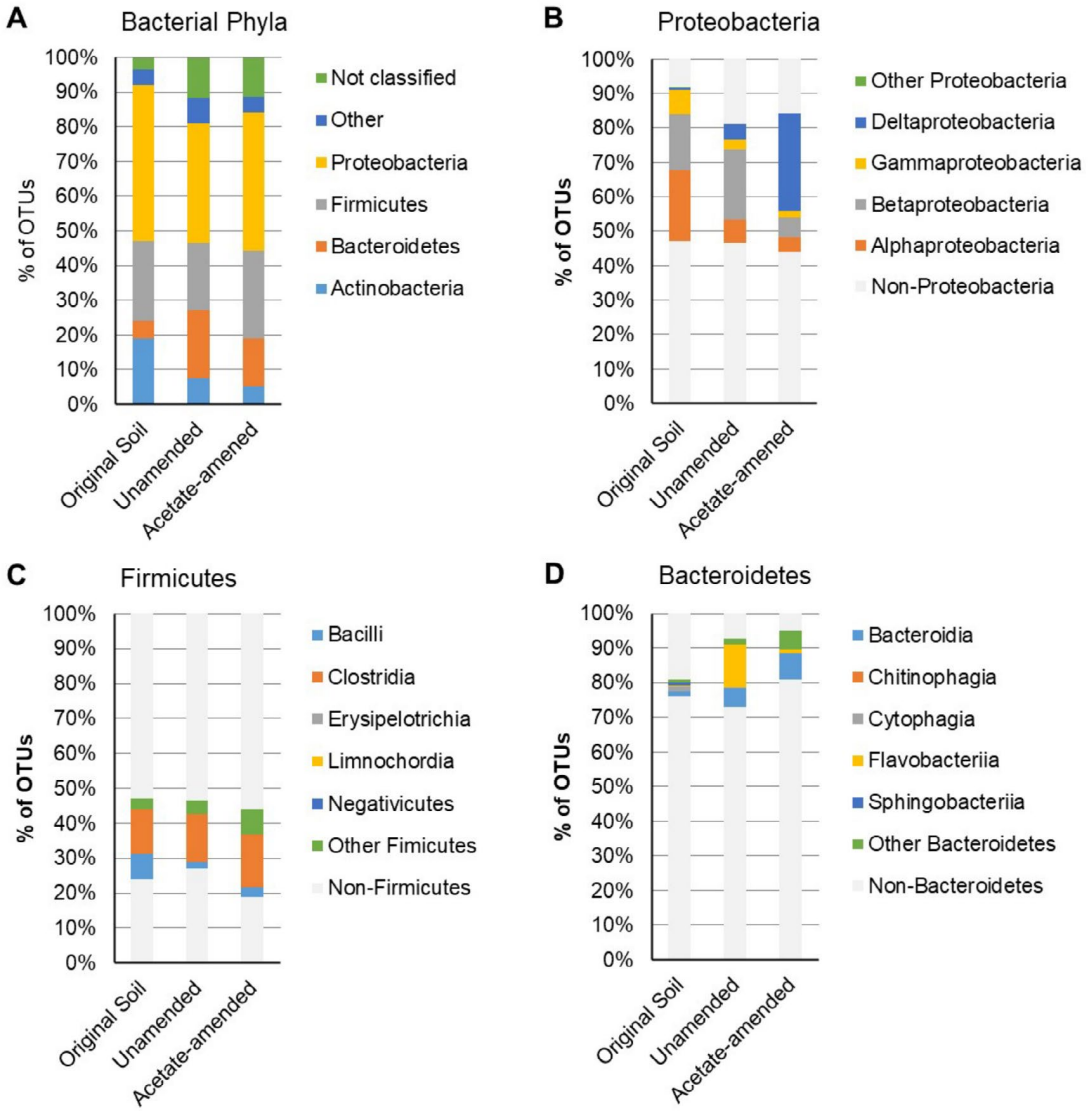
Phylogenetic diversity in the original soil and at the final sampling point in the unamended and acetate‐amended microcosm experiments determined by 16S rRNA gene sequencing: (A) distribution by phylum, and distribution by class within the (B) *Proteobacteria*, (C) *Firmicutes* and (D) *Bacteroidetes*.

On average, 88% ± 2% (unamended), 89% ± 1% (acetate‐amended) and 96% ± 1% (original soil) of 16S rRNA gene reads were assigned to a bacterial phylum with a confidence > 0.7 (Figure 2). Reads from the unamended microcosms were dominated by *Proteobacteria* (35% ± 2%), *Bacteroidetes* (20% ± 4%), *Firmicutes* (19% ± 2%) and unclassified (12% ± 2%). Reads from the acetate‐amended microcosms were dominated by *Proteobacteria* (40% ± 4%), *Firmicutes* (25% ± 2%), *Bacteroidetes* (14% ± 1%) and unclassified (11% ± 1%). Whereas reads from the original soil were dominated by *Proteobacteria* (45% ± 5%), *Firmicutes* (23% ± 4%), *Actinobacteria* (19% ± 2%) and *Bacteroidetes* (5% ± 1%).

The OTU richness (*D*_0_^*α*^), and the number of common OTUs (*D*_1_^*α*^) and dominant OTUs (*D*_2_^*α*^) were slightly lower in the unamended (611 ± 34, 74 ± 10, 23 ± 4) than in acetate‐amended microcosms (616 ± 47, 95 ± 24, 35 ± 16), which in turn were slightly lower than in the original soil (*D*_0_^*α*^ = 737 ± 62, *D*_1_^*α*^ = 121 ± 29, *D*_2_^*α*^ = 46 ± 12; Table S4).

Bray‐Curtis beta diversity values for dissimilarity between the replicates indicate that the unamended replicates were quite similar to each other at the level of OTUs (BC = 0.23, Table S5), as were the acetate‐amended replicates (BC = 0.27), with only slightly greater dissimilarity between the original soil replicates (BC = 0.32) There was greater dissimilarity between the unamended and acetate‐amended microcosms (BC = 0.56). Both the unamended and the acetate‐amended microcosms exhibited a greater dissimilarity from the original soil at the level of OTUs (BC = 0.63 and 0.70, respectively; Bray‐Curtis values range from 0 for populations with the same composition to 1 for populations that do not share any OTUs).

The decrease in the proportion of *Proteobacteria* in the two microcosm systems relative to the original soil is due to larger changes in the proportion of the dominant classes within the phylum. In the unamended system, there was a decrease in the proportion of alpha (from 21% ± 4% to 7% ± 0.7%), a modest decrease in the proportion of gamma (from 7% ± 1% to 3% ± 0.6%), together with a modest increase in the proportions of delta (from 1% ± 0.4% to 4% ± 1%) and beta (from 16% ± 3% to 20% ± 2%), relative to the original soil. In the acetate‐amended system, there was a marked decrease in the proportions of *Alpha‐, Beta‐ and Gamma‐proteobacteria* (from 21% ± 4% to 4% ± 0.3%, from 16% ± 3% to 6% ± 0.4% and from 7% ± 1% to 2% ± 0.3%, respectively), together with a marked increase in the proportion of *Deltaproteobacteria* (from 1% ± 0.4% to 28% ± 5%), relative to the original soil (Figure 2B).

The increase in the overall proportion of *Bacteroidetes* in the unamended microcosms relative to the original soil reflects a large increase in the proportion of OTUs classified as Flavobacteriia and a smaller increase in the proportion of OTUs classified as Bacteroidia (the former increase from 0.2% ± 0.1% to 12% ± 4% and the later from 1% ± 0.3% to 5% ± 1%). Whereas the increase in the overall proportion of *Bacteroidetes* in the acetate‐amended microcosms relative to the original soil reflects a larger increase in the proportion of OTUs classified as Bacteroidia and a modest increase in the proportion of OTUs classified as other *Bacteroidetes* (the former increase from 1% ± 0.3% to 7% ± 0.5% and the later from 1% ± 0.4% to 5% ± 2%) (Figure 2D).

The modest changes in the overall proportion of *Firmicutes* in the two microcosm systems relative to the original soil principally reflect a decrease in proportion of OTUs classified as bacilli (from 7% ± 2% to 2% ± 0.3% and 3% ± 0.4% in the unamended and acetate‐amended systems, respectively, Figure 2C).

### 3.3 The Impact of Acetate on Global Gene Expression in the Microcosms

The meta‐transcriptome data set comprised 53,019 genes, 24,974 from the unamended microcosm and 28,045 from the acetate‐amended microcosm (Table S8). Fifty six percent (29,712) genes comprising 12,922 genes in the unamended and 16,790 genes in the acetate‐amended samples could not be classified beyond the classification ‘bacteria’. A full breakdown of classification by taxa based on the MAGs derived from the whole genome sequencing is presented in Table S9.

**FIGURE 3:**
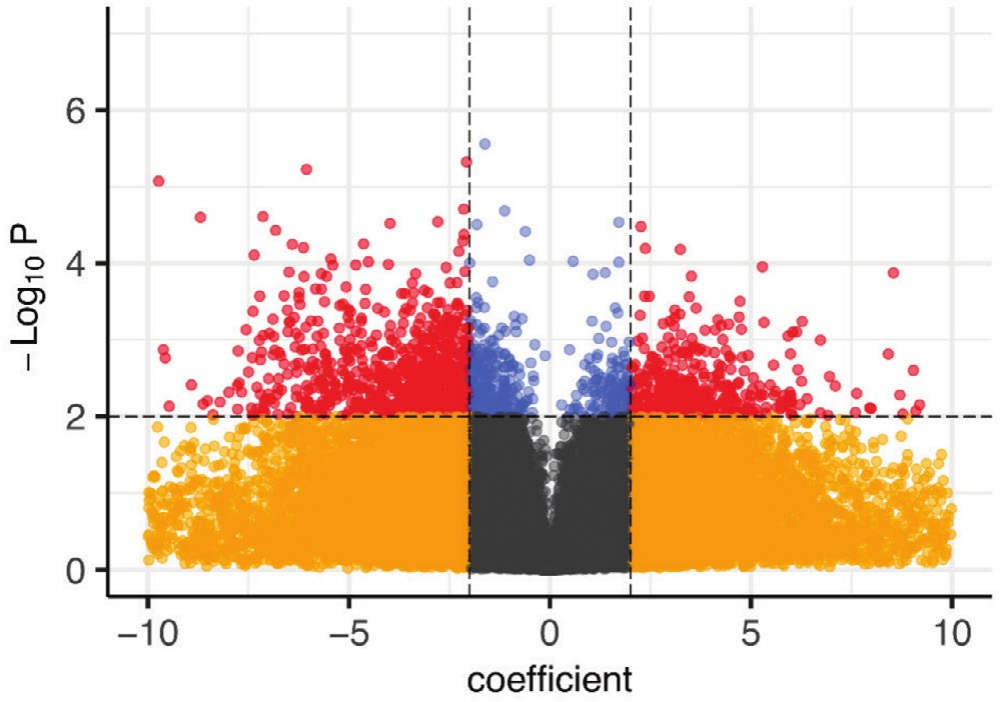
Differential expression analysis of transcripts found in both systems (acetate‐amended system B/unamended system A). Negative expression coefficient means upregulation in the unamended system, whereas positive ones mean upregulation in the acetate‐amended system. Red and blue dots show the 1534 differentially expressed genes (DEGs) where *p* value < 0.01. The red dots identify the subset of those DEGs where the |coefficient| is also > 2 (1127 of 1534 DEGs). Yellow and grey dots show transcripts where differential expression was not significant (51,485 transcripts).

The data set was analysed to identify genes that are differentially expressed between the unamended and the acetate‐amended populations and displayed on a volcano plot (Figure 3). Of the 53,019 transcripts in the data set, 1534 transcripts (~3%) were differentially expressed with a significance of *p* < 0.01 (−log_10_ *p* value of 2 shown by horizontal line in Figure 3). The left‐hand side of the volcano plot (negative coefficient) shows the transcripts which are more abundant in the unamended microcosms, whereas the right‐hand side (positive coefficient) shows transcripts which are more abundant in the acetate‐amended microcosms. Inspection of the list of 1534 DEGs (*p* < 0.01; Table S10) revealed 489 transcripts that were more abundant in the population in the acetate‐amended microcosm and 1045 which were less abundant. The differentially expressed list includes many transcripts for hypothetical proteins, which may encode functions required in this highly specialised environment that have not been well characterised previously.

**FIGURE 4:**
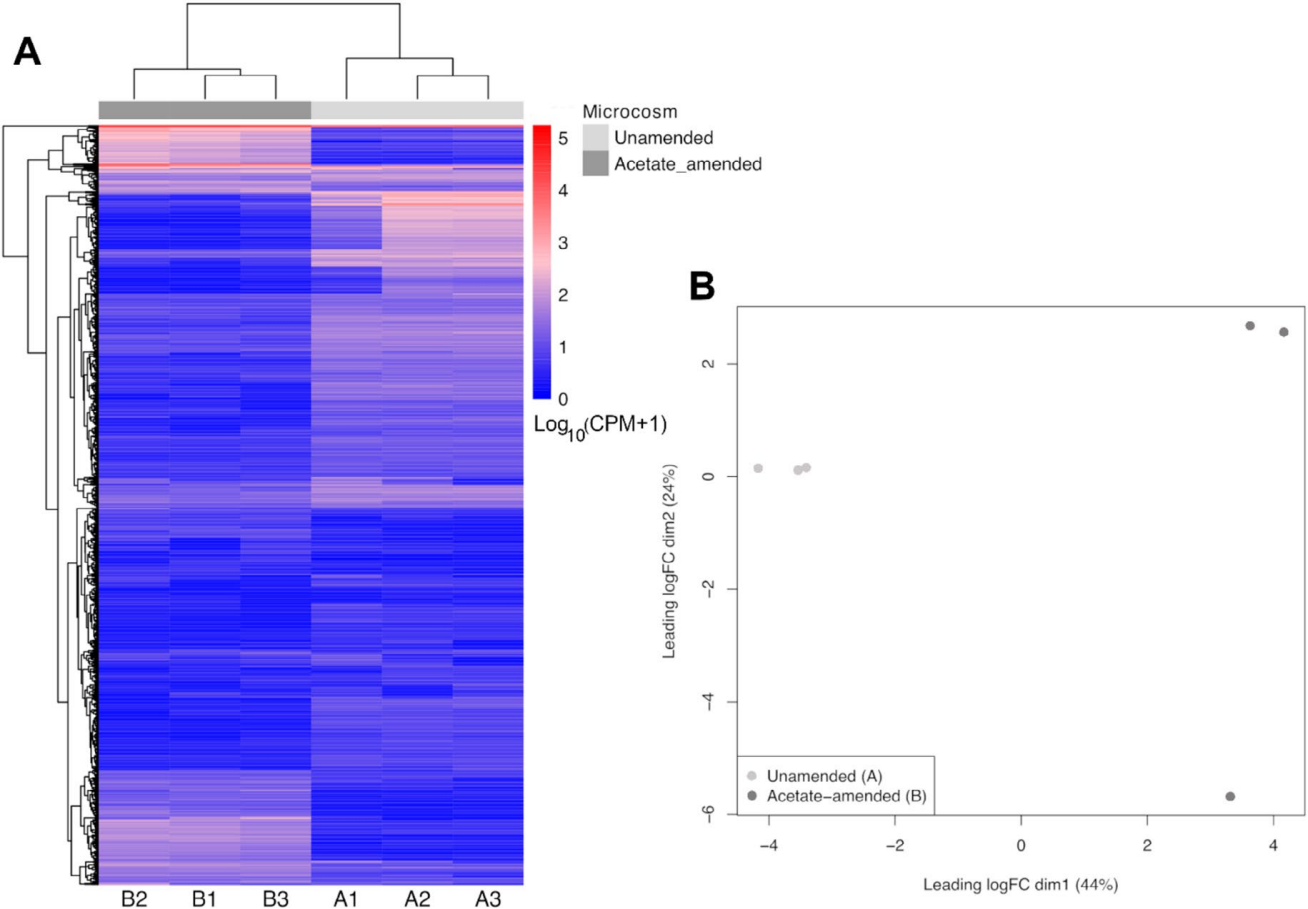
Unamended and acetate‐amended systems' gene expression profile. (A) Normalised expression (counts per million—CPM) heatmap of all 1534 DEGs depicting two clear sample clusters (columns' dendrogram) through unsupervised hierarchical clustering (HC) assessment. (B) Unsupervised multi‐dimensional scaling (MDS) plot of the ReadCounts of all 53 k genes across the six samples, corroborating the presence of both sample clusters observed from HC in panel A under a 44% explained variance on the first dimension. Replicates from the unamended system are A1, A2 and A3; replicates from the acetate‐amended system are B1, B2 and B3.

Hierarchical clustering was applied over counts per million (CPM) from these 1534 differentially expressed transcripts and displayed as a heat map (Figure 4A). The dendrogram of the rows shows similar patterns of response clustered into four gene groups: (1) strongly upregulated in the acetate‐amended microcosm compared to the unamended; (2) strongly downregulated in the acetate amended compared to the unamended microcosm; (3) weakly upregulated in the unamended microcosms; and (4) weakly upregulated in the acetate‐amended microcosm compared to the unamended microcosm. The dendrogram of the columns shows that biological replicates from each set of microcosms clustered accordingly.

A principal component analysis‐like MDS using all 53,019 genes that had read counts assigned to them (Figure 4B) corroborated the hierarchical clustering from the heat map (Figure 4A), showing that the acetate amended and unamended biological replicates clearly separated within dimension 1 (logFC) explaining 44% of the variance. One replicate in the acetate‐amended system (B2) separates from the other two replicates in Dimension 2 but remains clearly distinct from the replicates of the unamended system. This is also consistent with the hierarchical clustering, with the other two replicates of the acetate‐amended system being more similar to one another than to B2 (Figure 4A).

### 3.4 Insights Into Metabolic Processes Inferred From GO and KEGG Terms

**FIGURE 5:**
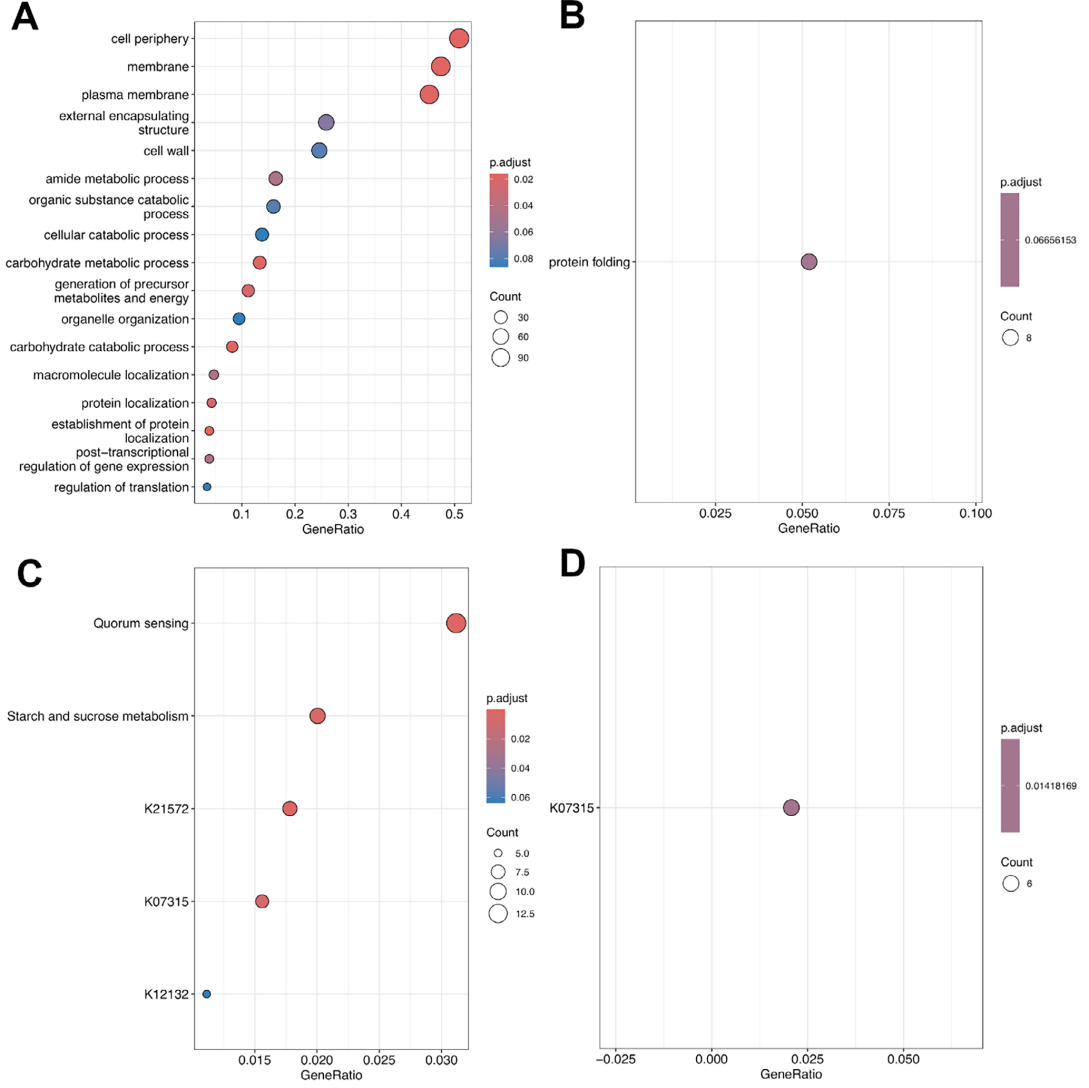
GO‐ and KEGG‐based gene enrichment analyses on the cluster of orthologous groups (COGs) derived from DEGs. (A) GO terms enriched in the unamended system, (B) GO term enriched in the acetate‐amended system, (C) KEGG pathways and orthologies enriched in the unamended system, (D) KEGG orthology enriched in the acetate‐amended system.

To interpret these gene expression changes in terms of biological processes and metabolic pathways taking place in the bacterial populations in the two microcosm series, clusters of orthologous genes (COGs) were assembled (as described in the materials and methods, and inspired by Vannier et al. 2023) and used to identify significantly enriched GO terms and Kyoto Encyclopaedia of Genes and Genomes (KEGG) pathways. Figure 5A shows GO terms that are significantly enriched in the population of the unamended microcosms. These relate particularly to processes involving the cell wall, cell periphery, plasma membrane and encapsulating structures as well as catabolic processes, especially carbohydrate metabolism. In contrast, only one GO term was significantly enriched in the population from the acetate‐amended microcosms (Figure 5B); protein folding.

KEGG pathways‐based enrichment analysis (Figure 5C) confirms the significance of carbohydrate metabolism in the populations of the unamended microcosms. KEGG Orthology accession K21572 points to SusD, which is a starch binding protein involved in the import of starch oligosaccharides into the periplasm (Shipman et al. 2000). There are multiple transcripts for *sus*D and its interaction partner (a putative maltodextrin porin) *sus*C in the list of differentially expressed transcripts.

KEGG Orthology K12132 (PrkC, StkP), which is also enriched in the unamended system, is a protein kinase implicated in developmental responses such as cell division, sporulation, and spore germination and biofilm formation (Madec et al. 2002; Shah et al. 2008; Beilharz et al. 2012). Quorum sensing also appears as an enriched pathway compared to the acetate microcosms. Responses can include regulation of growth, antibiotic resistance, heavy metal resistance and biofilm formation (Qu et al. 2024).

### 3.5 Changes in σ Factor Regulation

**FIGURE 6:**
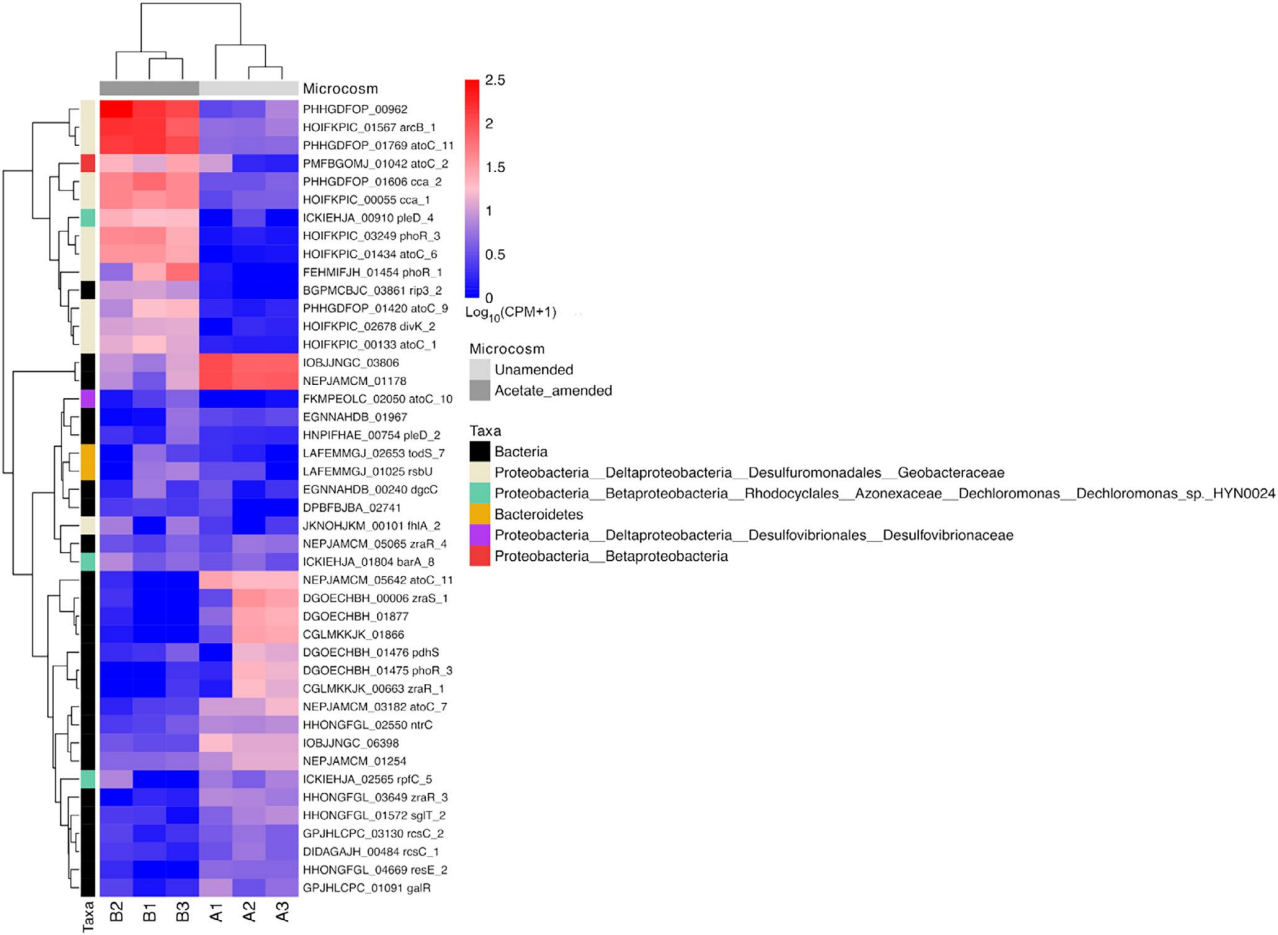
Heatmap of genes assigned to the phosphoserine phosphatase rsbU/P K07315 KEGG Orthology group showing differential expression according to taxa. Replicates from the unamended system are A1, A2 and A3; replicates from the acetate‐amended system are B1, B2 and B3. See text for details.

Interestingly, K07315 is enriched in both unamended and acetate‐amended microcosms (Figure 5C,D). K07315 describes a phosphoserine phosphatase RsbU/P which functions in transcriptional regulation through regulation of the RNA polymerase σ B subunit which provide the specificity for promoter selection (Yang et al. 1996; Bonilla 2020). Closer inspection of K07315 COGs' composition in both microcosms reveals differences in quantity and specificity, which indicate different responses of distinct bacterial populations to the two conditions. Taxonomical analysis of the transcripts assigned to K07315 indicates *Proteobacteria*, and particularly *Deltaproteobacteria*, respond differently in acetate‐amended samples (Figure 6). In contrast, another group of taxa, including *Bacteroidetes*, show little change between the two systems while a third group show the reverse response with higher transcript abundance in the unamended system. Most of these are classified as ‘bacteria’ indicating that further classification is not possible without additional information. Some of these changes can be explained by the changing abundance of the different taxa in the different systems as described by the 16S rRNA sequencing data (Figure 2) and MAG data (Table S9), so one must be cautious about ascribing these to changes in gene regulation rather than organism abundance.

The list of 1534 significantly differentially expressed transcripts (Table S10) contains 13 entries that are related to σ or anti‐σ factors (Table 1). Transcripts for Group 3 and 4 σ70s along with σ54s are more abundant in the acetate‐amended microcosms and predominantly arise from *Geobacter* (Group 3 and σ54) and unclassified bacteria (Group 4) (see Table 1). σ factors are regulated post translationally through multiple mechanisms, including sequestration by binding to anti σ factors (Österberg et al. 2011). Five anti‐σ factors show reduced transcript abundance in the acetate‐amended microcosms. Anti‐σ factor *rgs*l is the most strongly reduced and is involved in transmitting extracellular polysaccharide sensing signals to activate degradation pathways via SigI (Chen et al. 2023). RbsV is part of the σ/anti‐σ regulatory circuit, which involves K07315 (Table 1, Figure 5C,D).

**TABLE 1:** Differentially regulated σ factors and anti‐σ factors (positive values of the coefficient indicates that a gene is upregulated in the acetate‐amended system).

Gene ID	Coef.	*p*	Gene description	Synonym	Group	KEGG ID	Taxa
DGOECHBH_02183	−5.94	0.0006	Anti‐σ‐I factor RsgI2				Bacteria
CGLMKKJK_01354	−5.90	0.0013	Anti‐σ‐I factor RsgI2				Bacteria
BCAGEAMG_00358	−4.69	0.0012	RNA polymerase σ factor RpoD	housekeeping σ70	1	K03086	Bacteria; Proteobacteria; Deltaproteobacteria; Desulfuromonadales
DGOECHBH_01741	−4.65	0.0023	Anti‐σ‐I factor RsgI3				Bacteria
CGLMKKJK_01361	−3.58	0.0004	Anti‐σ‐I factor RsgI2				Bacteria
GHNPANGO_03264	−3.05	0.0017	rsbV_2Anti‐σ‐B factor antagonist			K04749	Bacteria
DPBFBJBA_00149	−1.65	0.0053	ECF RNA polymerase σ factor SigW		4	K03088	Bacteria
HOIFKPIC_01269	2.35	0.0052	RNA polymerase σ factor FliA	Sig28, SigF	3	K02405	Bacteria; Proteobacteria; Deltaproteobacteria; Desulfuromonadales; Geobacteraceae
IOBJJNGC_00907	2.52	0.0044	ECF RNA polymerase σ factor SigE	rpoE	4	K03088	Bacteria
NEPJAMCM_05121	2.65	0.0018	ECF RNA polymerase σ factor SigE	rpoE	4	K03088	Bacteria
HOIFKPIC_00402	2.90	0.0040	RNA polymerase σ factor RpoH	Sig32	3	K03089	Bacteria; Proteobacteria; Deltaproteobacteria; Desulfuromonadales; Geobacteraceae
HOIFKPIC_01620	4.29	0.0067	rpoN RNA polymerase σ54 factor	rpoN		K03092	Bacteria; Proteobacteria; Deltaproteobacteria; Desulfuromonadales; Geobacteraceae
PHHGDFOP_00740	4.40	0.0080	rpoN RNA polymerase σ54 factor	rpoN		K03092	Bacteria; Proteobacteria; Deltaproteobacteria; Desulfuromonadales; Geobacteraceae

*Note:* σ factors provide *promoter* selectivity to core RNA polymerase. Bacterial species have multiple σ factors with different synonyms, but apart from σ54's they are all members of the σ70 family.

### 3.6 Transcripts of Acetate Metabolism

**FIGURE 7:**
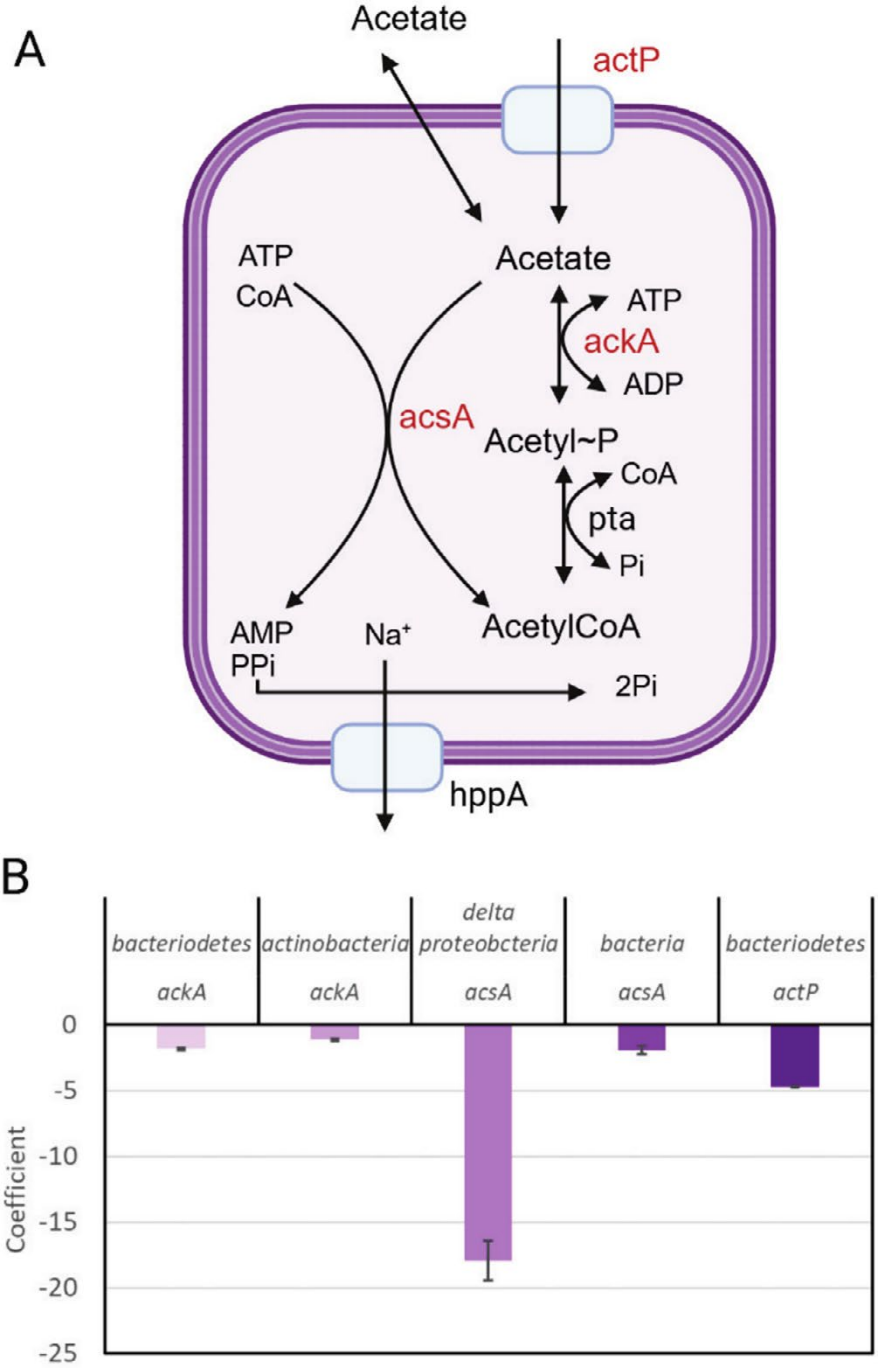
Transcripts associated with acetate metabolism in bacteria. (A) schematic diagram showing pathways of acetate assimilation/dissimilation. Transcripts of genes shown in red are displayed in the graph in B. (B) changes in transcript abundance between the acetate amended and unamended microcosms. Only transcripts showing significant differential expression (*p* < 0.01) are shown. Negative coefficients mean lower transcript abundance in the acetate‐amended microcosm compared to unamended microcosm. Created in BioRender. Baker (2025) https://BioRender.com.

Acetate can be assimilated by microbes as a carbon source or can be excreted as a waste product (Figure 7A). Cell membranes are freely permeable to acetate, so a transporter is not required, but ActP (YcjG) which shows reduced expression in the acetate‐amended microcosms (Figure 7B), may be important in scavenging low levels of acetate from the environment.

In dissimilatory acetate metabolism, acetyl CoA is converted to acetyl phosphate (acetyl~*P*) via phosphotransacetylase (Pta) and acetyl~*P* is converted to acetate with the production of ATP by acetate kinase (AckA). This allows substrate‐level phosphorylation to produce ATP under anaerobic conditions (Figure 7A). The AckA‐Pta pathway can also assimilate acetate but only when it is present in the mM range. Two acetate kinases, HMEJDMKA_01753 (*Bacteroidetes*) and LDFLFJEJ_01416 (*Actinobacteria*), show a small decrease in abundance in the acetate‐amended microcosms while *pta* is not differentially expressed. Both taxa show a decrease in abundance in the acetate‐amended microcosms (Figure 2 and Table S9). In the absence of acetate, these taxa may be scavenging low quantities of acetate released by other organisms via ActP and using dissimilatory acetate metabolism for ATP production.

AMP‐ACS (AcsA *K*_*m*_ for acetate ~200 μM) assimilates acetate at low concentrations (Wolfe 2005). Two *acs*A transcripts were differentially expressed (*p* < 0.01): (i) OFLLCJLC_03493 (*Deltaproteobacteria*) was very strongly reduced in abundance (−17.9) in the acetate‐amended microcosms, despite the large increase in the proportion of *Deltaproteobacateria* in this system compared to the unamended system (Figure 2 and Table S9); and (ii) IOBJJNGC_01071 (‘bacteria’) was also reduced in abundance (−1.9) in the acetate‐amended microcosms (Figure 7B).

### 3.7 Transcripts of Sulphur Metabolism

Only two transcripts for genes associated with dissimilative sulphate reduction are among 1534 transcripts that differentially regulated in the two systems (*p* < 0.01, |coefficient| > 2). Both are for the gene *apr*A (KEGG Orthology K00394). One transcript, PMFBGOMJ_01722 (*Betaproteobacteria*), is strongly downregulated in the acetate‐amended system. The other, BKGNLCHP_01161 (‘bacteria’) is upregulated in the acetate‐amended system (Table S10).

## 4 Discussion

In this paper we report the effects of acetate stimulation on the meta‐transcriptome of microbial populations in microcosms established from soil samples taken from below a previously described COPR ore disposal legacy site (Stewart et al. 2024) and periodically challenged with Cr. Both acetate amended and unamended microcosms are stressed environments due to alkaline pH, osmotic stress and metal contamination. Concurrent geochemical measurements, 16S RNA sequencing and metagenomic data allow us to link population changes, geochemical processes and gene expression to provide insights into the response of these populations to the addition of an electron donor.

### 4.1 Dominant Geochemical Processes at the Point of Sampling

About half of the microbially available iron was initially in the reduced Fe(II) oxidation state and the acetate‐amended system progressed to the point where sulphate reduction was commencing, whereas the unamended system was still poised at a point where iron reduction was the dominant dissimilatory process at the time of sampling. The transcriptome dataset indicates there was a no clear difference in the behaviour of the bacterial populations with respect to sulphur metabolism. Perhaps sulphur metabolism was not yet a dominant metabolic process (unlike our previous study: Stewart et al. 2024), so it did not produce a large difference between the two transcriptomes, or possibly that early changes in sulphur metabolism are predominantly post‐transcriptional and not captured by transcriptomics.

Cr(VI) was very quickly removed from solution at the start of the microcosm experiments, most probably by reductive precipitation as Cr(III) by Fe(II) oxidation to Fe(III) (Buerge and Hug 1999), but without much decrease in the proportion of the microbially available iron present as Fe(II) as it was available in excess. Subsequent Cr(VI) spikes were similarly removed very quickly. The microbial populations showed similar shifts in both studies, especially the increase in *Deltaproteobacteria* (particularly *Geobacter* spp.) in the acetate‐amended system at the expense of *Bacteroidetes* (Flavobacteria). This independent replication lends confidence to the observation of Williams et al. (2011) that *Geobacter* spp. dominated the microbial community in groundwater in an acetate biostimulation field experiment at the point where sulphur reduction and U(VI) removal were occurring.

### 4.2 Changes in Metabolic Processes in Response to Acetate

The meta‐transcriptome data showed a profound shift in metabolic processes when acetate was provided to the microcosms. Processes associated with the assimilation of complex carbohydrate sources and at the cell/environment interface (GO terms cell periphery/membrane/plasma membrane/cell wall/external encapsulating structure) were all downregulated. It is interesting that several such processes are regulated by acetyl phosphate (acetyl ~*P*), an intermediate of acetate metabolism, which acts as a global signalling molecule in bacteria via phosphorylation of two component system response regulators (RRs). These include chemotaxis (Wolfe 2005), biofilm development (Wolfe et al. 2003) and flagella and capsular biosynthesis (Fredericks et al. 2006).

Acetyl phosphate increases under conditions of starvation and/or lack of oxygen (Wolfe et al. 2003) and promotes expression of genes associated with Type I pilus assembly, capsule biosynthesis, biofilm formation and some stress effectors (Wolfe et al. 2003). While both microcosm series are anaerobic, the unamended system is more carbon limited than the acetate‐amended system, relying on the residual soil organic carbon content at the start of the experiment (3 wt%) compared to 48 mM acetate at the point of sampling for the acetate‐amended system. Therefore, the expression of genes related to pilus assembly, capsule biosynthesis and biofilm formation might be expected to be higher in the unamended system, which is reflected in the enrichment in GO terms related to these processes in the unamended compared to the acetate‐amended microcosms.

Another mechanism of regulation of many of these processes is via expression or activity of different σ factors (Table 1). Bacterial species have multiple σ factors with different synonyms, but apart from σ54's they are all members of the σ70 family. σ70 proteins are divided into four groups, Group I are the ‘housekeeping’ σs, Group 2 are generally dispensable for growth, but co‐ordinate expression of stationary phase/stress functions. Group 3 tend to regulate developmental checkpoints and Group 4 respond to signals from the extra cytoplasmic environment, and to Fe deficiency. A distinct class of σ factors, σ54's, co‐ordinate diverse physiological functions including utilisation of alternative carbon sources, nitrogen assimilation and assembly of motility organs (Österberg et al. 2011). σ factors provide the specificity to RNA polymerase for promoter recognition, therefore co‐ordinate transcription of specific subsets of genes under general stress conditions (Bonilla 2020; Bouillet et al. 2024). In *B. subtilis* σB is an alternative σ factor which binds to core RNA polymerase to mediate general stress responses which cells experience in a slowly growing or non‐ growing state. σB is activated by (1) energy or nutritional stress, by entry into stationary phase and (2) environmental stress (e.g., osmotic stress) through the activity of anti‐σ factors (Hughes and Mathee 1998; Bonilla 2020). Activity of σ factors is regulated through a pathway involving RbsU (K07315) which is important for transmitting environmental stress and dephosphorylates RbsV (Table 1) which complexes the kinase RbsW, which is an anti‐σ factor, allowing σB to initiate transcription under conditions where RbsU is more active. Thus, the DE of σ‐ and anti‐σ factors represents adaptive mechanisms of microbial populations under multiple stresses.

While there are a wealth of studies reporting the transcriptional responses of individual bacterial species in batch or continuous culture to acetate, it is difficult to compare results to the complex and shifting microbial communities found in contaminated environmental samples. The limited studies on Cr(VI) remediation have focussed on responses to the metal itself (Pei et al. 2020), rather than on stimulating metabolic capability to enhance bioremediation potential, as in this study. Lara et al. (2021) studied the transcriptome of *Klebsiella* spp. adapted to Cr(VI) under aerobic conditions and reported upregulation of alternative σ factors (*fec*l *rpo*S and *rpo*E in *Klebsiella*), downregulation of energy and carbohydrate metabolism, and upregulation of envelope and osmotic stress responses in response to Cr(VI) stress. However, the geochemical data reported in Figure 1 and (Stewart et al. 2024) show that Cr(VI) is rapidly removed from solution in both microcosms and therefore cannot be the reason for the differential gene expression we observe in the acetate amended compared to the amended microcosms. These responses could represent more general stress responses (Bonilla 2020) which are partially alleviated by the provision of an excess of acetate: a readily metabolised carbon source and electron donor.

### 4.3 Meta‐Transcriptome‐Based Insights Into Microbial Life History Strategies

Microbial communities are shaped by the life history strategies adopted by their members. These are defined by a set of traits that correlate due to either evolutionary or physiological trade‐offs. Different community members may adopt different strategies to maximise their survival under fluctuating conditions. Malik et al. (2019) consider life history strategies in the context of soil organic carbon cycling and define three competing life history strategies: growth yield (Y; biomass production per unit resource), resource acquisition (A; degradation of complex substrates, uptake of simple substrates), and stress tolerance (S; damage repair, maintenance of cellular integrity). When resources are abundant and conditions are ambient, the Y strategy is preferred. When resources become scarce, A will be preferred. Under stressed conditions, S is preferred. The microcosms can be considered as stressed systems, with the unamended microcosms being in addition resource limited. Our data provide evidence that acetate addition resulted in a reduction of resource acquisition functions in the community, and the 16S rRNA and MAG data also support the idea that different taxa invested in different strategies—for example, population growth in the *Deltaproteobacteria* in the presence of acetate. The K07315 heatmap suggests both populations are stressed, which again is not surprising. Measurements for yield are quite challenging to extract from ‘Omics datasets’ (Malik et al. 2019). However, expression of the ribosomal protein encoding gene *rps*C was reported to be a good marker for growth of *Geobacter* spp. in an environmental system (Holmes et al. 2013). In our data set, transcript FKMPEOLC_00462, which encodes a putative RpsC from *Desulfovibrionaceae* (*Deltaproteobacteria*) is strongly (5.19) upregulated in the acetate‐amended microcosms. This is consistent with the increased abundance of this taxa in this microcosm series as inferred from the 16S rRNA gene sequencing.

In summary, this study combined geochemical, 16S rRNA gene sequencing and meta‐transcriptomics approaches to investigate microbial communities in microcosms established from soil samples derived from below a legacy COPR disposal site. The data provide an insight into the biochemical and physiological processes taking place within these microbial populations, which can, in part, be explained by current understanding of how acetate regulates microbial gene expression at a mechanistic level. Linking results from laboratory and field is challenging due to the complex and highly variable conditions experienced in the field; for example, the expression of *apl*A, B and C (ActP hologues in *Geobacter* spp.; Williams et al. 2011) is correlated with acetate level in continuous culture, but this breaks down under field conditions (Elifantz et al. 2010). Microcosms, while not fully representative of field conditions, can be a useful approach to help bridge the gap between laboratory and field scale studies. The behaviour of the populations within the two microcosms is consistent with the model proposed for trait‐based microbial life history strategies (Malik et al. 2019).

## Author Contributions

**Douglas I. Stewart:** conceptualization, formal analysis, funding acquisition, investigation, visualization, writing – original draft, writing – review and editing. **Elton J. R. Vasconcelos:** conceptualization, data curation, formal analysis, visualization, writing – review and editing, software. **Ian T. Burke:** conceptualization, investigation, resources, writing – review and editing. **Alison Baker:** conceptualization, formal analysis, investigation, visualization, writing – original draft, writing – review and editing.

## Ethics Statement

The authors have nothing to report.

## Conflicts of Interest

The authors declare no conflicts of interest.

## Supporting information


**Data S1.** emi470148‐sup‐0001‐supinfo.

**Table S7.** Properties of the metagenome‐assembled genomes (MAGs) from the microcosms.

**Table S8.** List of all genes from the MAGs from the unamended and aetate‐amended systems that had RNA read counts assigned to them and assessed by MTX model algorithm for differential expression. The letter A indicates the unamended system the letter B indicates the acetate‐amended system.

**Table S9.** Rank summary of taxa associated to all MTX model‐assessed genes from the MAGs. The letter A indicates the unamended system the letter B indicates the acetate‐amended system.

**Table S10.** List of 1534 differentially expressed genes (DEGs) identified by MTX model (*p* value < 0.01).

## Data Availability

The data that support the findings of this study are openly available in the European Nucleotide Archive (ENA) at https://www.ebi.ac.uk/ena/browser/view/PRJEB51999, reference number PRJEB51999.
