# Supplementary material for: Meta‐Transcriptomes From Microcosms From a Cr Impacted Soil Provides Insights Into the Metabolic Response of the Microbial Populations to Acetate Stimulation
Source: Environ Microbiol Rep. 2025 Jul 7;17(4):e70148. doi: 10.1111/1758-2229.70148 (PMC12234377; doi:10.1111/1758-2229.70148)
Supplement: Supplementary file 1 — Data S1. emi470148‐sup‐0001‐supinfo. [file EMI4-17-e70148-s001.docx]

Supplementary Information for:

Meta-transcriptomes from microcosms from a Cr impacted soil provides insights into the metabolic response of the microbial populations to acetate stimulation.

Douglas I. Stewart^1^*, Elton J. R. Vasconcelos^2^, Ian T. Burke^3^, Alison Baker^4^.

^1^ School of Civil Engineering, University of Leeds, Leeds LS2 9JT, UK (d.i.stewart@leeds.ac.uk)

^2^ Leeds Omics, University of Leeds, Leeds LS2 9JT, UK (E.Vasconcelos@leeds.ac.uk)

^3^ School of Earth and Environment, University of Leeds, Leeds LS2 9JT, UK (i.t.burke@leeds.ac.uk)

^4^ School of Molecular and Cellular Biology, University of Leeds, Leeds LS2 9JT, UK (a.baker@leeds.ac.uk)

* Corresponding author:

Table S1. Mineralogy of the grey clay determined by relative intensity ratio (RIR) quantitative x-ray diffraction analysis (qXRD)

|  | Mineral % | | | | | | | |  |
| --- | --- | --- | --- | --- | --- | --- | --- | --- | --- |
| Sample Depth (m) | Quartz | Albite | Microcline | Calcite | Mica | Illite-smectite | Kaolinite | Chlorite | Total |
| 2.9 | 37.9 | 9.0 | 3.8 | 6.4 | 4.8 | 23.3 | 6.3 | 8.3 | 99.8 |

Table S2. Concentrations of selected trace elements present in the grey clay determined by XRF and LOI determined at 550°C.

|  | Element % | | | | | LOI |
| --- | --- | --- | --- | --- | --- | --- |
| Sample Depth (m) | Fe | Ca | Mn | S | Cr | % |
| 2.9 | 3.4 | 3.0 | 0.1 | 0.3 | 0.2 | 8.1 |

Table S3. Chemical composition of the ditch water.

|  | Cr(VI) | Cl^-^ | NO_3_^-^ | SO_4_^2-^ | pH |
| --- | --- | --- | --- | --- | --- |
|  | mmol.L^-1^ | | | |  |
| Ditch Water | 88 | 837 | 100 | 1038 | 8.8 |
| Conditioned Ditch Water | 420 | 837 | 100 | 1038 | 9.0 |

Table S4. Alpha diversity (Hill Numbers or order 0, 1, 2) where D_0_^a^ is the OTU richness, and D_1_^a^ and D_2_^a^ are measures of the number of common and dominant OTUs.

|  | D_0_^α^ | D_1_^α^ | D_2_^α^ |
| --- | --- | --- | --- |
| Original soil | 737 (±62) | 121 (±29) | 46 (±12) |
| Unamended | 611 (±34) | 74 (±10) | 23 (±4) |
| Acetate-amended | 616 (±47) | 95 (±24) | 35 (±16) |

Table S5. Bray-Curtis dissimilarity matrix as a measure of beta diversity between the samples (values range from 0 for populations with the same composition to 1 for populations that do not share any OTUs).

|  | Original  soil | Unamended | Acetate-amended |
| --- | --- | --- | --- |
| Original soil | 0.322 | 0.630 | 0.702 |
| Unamended |  | 0.230 | 0.562 |
| Acetate-amended |  |  | 0.266 |

Table S6. Software settings used for the metatranscriptomics data analysis

| **Software** | **Reference** | **Software parameters used** |
| --- | --- | --- |
| bowtie2 | Langmead and Salzberg (2012) | --sensitive-local -N 1 --no-mixed --no-discordant --no-unal |
| featureCounts | Liao et al. (2013) | -p -B -C -M -O --fraction -F GFF -t CDS . |
| MTXmodel | Zhang et al. (2021) | --min_abundance 1 --min_prevalence 0.33 --max_significance 0.25 --min_variance 0.0 --correction BH --standardize TRUE --normalization TMM --transform LOG --analysis_method LM --cores 8 --fixed_effects env --plot_heatmap TRUE --plot_scatter TRUE --reference env --rna_dna_flt strict --input_dnadata. |

Langmead, B. and Salzberg, S. L. (2012). Fast gapped-read alignment with Bowtie 2. Nature Methods, 9, 357-359.

Liao, Y., Smyth, G. K. and Shi, W. (2013). featureCounts: an efficient general purpose program for assigning sequence reads to genomic features. Bioinformatics, 30, 923-930.

Zhang, Y., Thompson, K. N., Huttenhower, C. and Franzosa, E. A. (2021). Statistical approaches for differential expression analysis in metatranscriptomics. Bioinformatics, 37, i34-i41.

**Tables S7, S8, S9 and S10 are in an excel file within the supplementary information.**

Table S7. Properties of the metagenome-assembled genomes (MAGs) from the microcosms.

Table S8. List of all genes from the MAGs from the unamended and aetate amended systems that had RNA read counts assigned to them and assessed by MTXmodel algorithm for differential expression. The letter A indicates the unamended system the letter B indicates the acetate amended system.

Table S9. Rank summary of taxa associated to all MTXmodel-assessed genes from the MAGs. The letter A indicates the unamended system the letter B indicates the acetate amended system.

Table S10. List of 1534 differentially expressed genes (DEGs) identified by MTXmodel (p-value < 0.01).


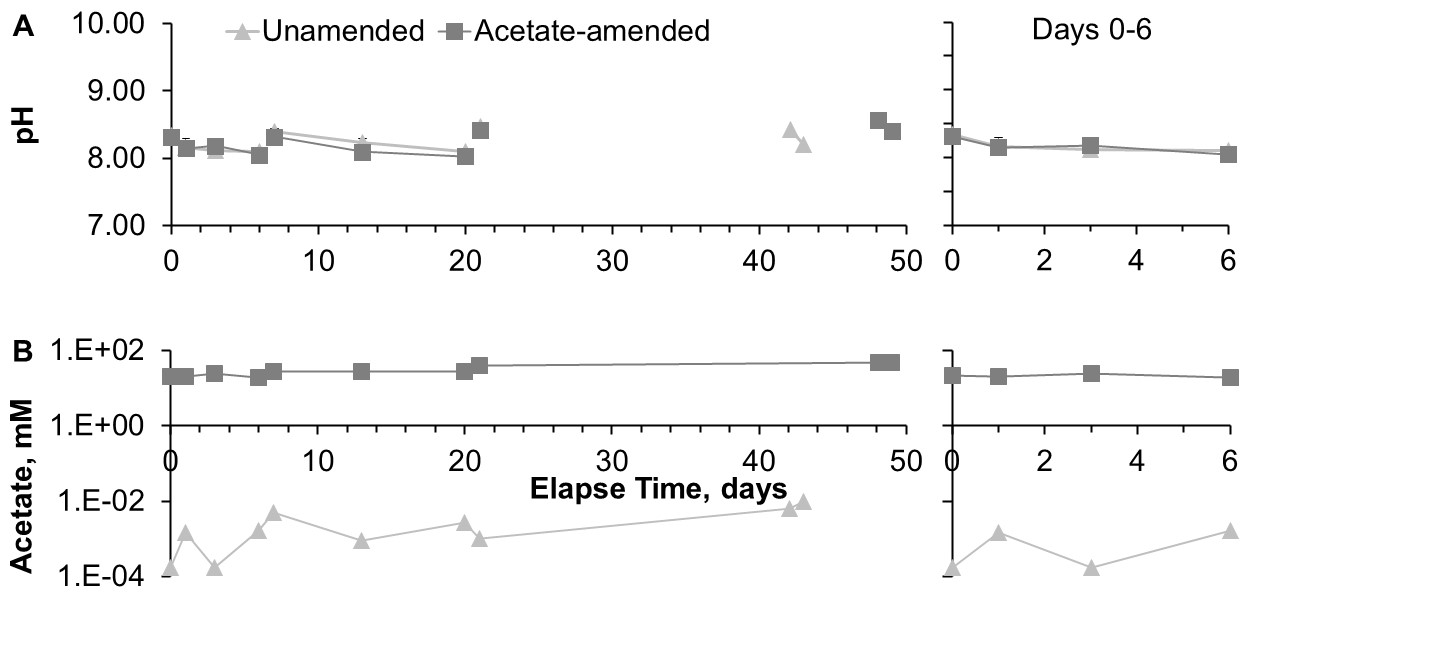


Figure S1. Geochemical conditions in the unamended and acetate-amended microcosms (A) pH vale and (B) acetate. Mean values from triplicate microcosms. Panels on right hand side show an expanded view of days 0-6.
